# The Transmission of Antibiotic Residues from Treated to Untreated Pigs and the Barn Environment

**DOI:** 10.3390/antibiotics15100977

**Published:** 2026-10-03

**Authors:** Larissa J. M. Jansen, Maura Arends, Laura L. M. van Asseldonk, Ron J. Berentsen, Gregg Roelofs, Daan Siebelink, Milou G. M. van de Schans

**Affiliations:** Wageningen Food Safety Research, Akkermaalsbos 2, 6708 WB Wageningen, The Netherlands

**Keywords:** antimicrobials, livestock production, prudent use, sulfadimidine, enrofloxacin

## Abstract

Background/Objectives: This study aimed to understand the extent of transmission of antibiotic residues from treated to untreated pigs and the barn environment using different pen set-ups. This is in order to create awareness and to stimulate prudent use of antibiotics in livestock farming. Methods: Transmission, expressed as the (relative) residue level, of sulfadimidine, enrofloxacin and its metabolite ciprofloxacin was determined using animal wipes, surface wipes and manure samples of pigs in four different compartments with variations in interconnected or distanced pens and type of pen partitions (open or closed). Results: Most antibiotic residues in untreated pigs were detected with a combination of open partitions and interconnected pens. Separating the pens with space in between minimized detectable antibiotic residues in the manure of untreated pigs, whereas interconnected pens did show antibiotic residues in internal matrices. Antibiotic residues were detected in the barn environment after treatment in all different pen set-ups. Conclusions: After treatment, transmission of antibiotic residues occurred in the barn environment and in the untreated pigs housed in the same barn. The spatial distribution of residues suggests that airborne dust and/or aerosols may be important routes of external transmission. Results under these experimental conditions indicate that restricting physical contact between treated and untreated pigs may be an important strategy for reducing antibiotic residues in internal matrices. This set-up also showed lower external residue levels, especially when closed partitions are used.

## 1. Introduction

Antibiotics are used to combat bacterial infections in animal husbandry. It is known, however, that the overuse of antibiotics could lead to selection of resistant bacteria, resulting in antibiotics no longer being effective. Antimicrobial resistance is directly responsible for 133,000 deaths and indirectly linked to 541,000 deaths each year in the European Union [1]. Reduction in antibiotics and their prudent use in animal husbandry are therefore of the utmost importance. 

Although antibiotic use has already been reduced drastically in many European countries, including the Netherlands [2], use remains inevitable, causing antibiotic residues to be present both in edible parts of livestock and in animal by-products, such as manure [3]. It is known that 70–90% of the antibiotic residues are excreted unchanged in their active form through urine and manure [4]. Through contaminated manure, antibiotic residues could be transferred from farm to field, creating another pathway towards food and humans [3,5,6]. This concern, among other things, has made antibiotic residues in the environment become a more widely researched topic. 

Aside from the transmission of antibiotic residues from farm to field, it has become apparent that antibiotic residues in the direct environment of livestock animals can expose untreated animals to antibiotic residues and can have an effect on the selection of resistant bacteria [7]. Hamscher et al. found during a retrospective study that a cocktail of antibiotics was still present in different dust samples collected from a piggery over two decades [8], indicating that these residues can persist in barn dust for a prolonged time. The presence of antibiotic residues in the barn also contributes to an increase in antimicrobial resistance, as seen in studies in European pig and poultry farms, where transmission of enrofloxacin leads to amplification and selection of resistant *Escherichia coli* (*E. coli*), and ceftiofur treatment influences the resistance patterns of intestinal *E. coli* in both treated and untreated pigs housed in the same barn [9,10,11]. These studies convey the importance of studying the transmission of antibiotic residues from treated to untreated animals and to reduce the spread in the barn environment during antibiotic treatment. 

There is a scarcity of research on the extent of transmission of antibiotic residues from treated to untreated animals, the barn environment and how to abate it. There are studies showing that using different types of oral feed can reduce transmission of residues or resistance genes [7,12] or that barn management conditions can reduce or prevent transmission of antibiotic residues to untreated animals [13]. However, to better understand the behaviour of antibiotic residues and the route(s) of transmission to untreated animals, a more systematic approach to study transmission itself is necessary. 

This research describes the results of a controlled animal study conducted with pigs in four separate compartments (1–4) with different pen arrangements: separated pens with (1) closed partitions and (2) open partitions, and interconnected pens with (3) closed partitions and (4) open partitions. It explored the internal (manure taken rectally) and external (animal wipes) residue transmission over time of sulfadimidine and enrofloxacin from treated pigs housed in one side of the compartment to untreated pigs in the other pens on the other side of the compartment. Additionally, in one of the compartments, urine from treated and untreated pigs within the same pen was taken. Furthermore, the transmission of residues to the direct environment was mapped by taking wipe samples of predefined surfaces above the pens and in the corners of the compartment. This study set-up provides insights into the transmission of antibiotic residues in different pen set-ups in the barn. The results help create awareness of the importance of limiting transmission of antibiotic residues, give first insights into reducing transmission to untreated animals and stimulate prudent use and treatment.

## 2. Results

### 2.1. Method Validation and Quality Control

After the compartments and pens were set up, but before the pigs arrived, a total of 84 wipe samples were taken from the PVC plates, fencing, feeding system and pipes above the pens and analyzed to ensure no detectable residues were present in the environment at the start of the study. No residues of enrofloxacin, ciprofloxacin or sulfadimidine were found above the LOQ. Furthermore, on the first day of sampling (day −4), no treatment with enrofloxacin or sulfadimidine had taken place yet. No residues of sulfadimidine, enrofloxacin or ciprofloxacin were detected above the LOQ for the animals in any compartment (Supplementary Materials Figures S1–S6).

The determined limit of quantification (LOQ) for all three antibiotics was 10 µg L^−1^ for urine and 10 µg kg^−1^ for manure. For wipes, the LOQ for sulfadimidine and ciprofloxacin was 5 ng per wipe, and for enrofloxacin, 1 ng per wipe could be achieved. For all matrices, no interfering signals were present at the retention time of the analytes, meaning that the selectivity is sufficient. In all cases, concentrations determined above the LOQ comply with the confirmation criteria (ion ratio and retention time) as stated in (EU) 2021/808 [14]. Quantitative parameters were determined in an in-house method validation using pig manure and complied with the applicable quantitative criteria. Trueness was 94% for ciprofloxacin, 110% for enrofloxacin, and 108% for sulfadimidine, while reproducibility was 6.6%, 8.3%, and 12%, respectively. For the urine method, quantitative parameters have not been established specifically for pig urine, but were previously determined in an in-house method validation using bovine urine. Trueness and reproducibility complied with the applicable quantitative criteria, with trueness ranging from 99 to 108% for ciprofloxacin, from 85 to 93% for enrofloxacin, and from 100 to 110% for sulfadimidine. Reproducibility ranged from 9.6 to 10.8%, from 9.4 to 14.9%, and from 7.0 to 9.1%, respectively. No quantitative parameters were determined for wipe samples, as the variability associated with wipe sampling, including differences in the sampled surface and the amount of material collected, was considered too high to support quantitative measurements. Overall, the analytical methods showed sufficient performance to support comparisons between samples and identify trends in residue concentrations.

### 2.2. Depletion of Antibiotics from Urine, Manure and Wipes in Treated and Untreated Pigs in the Same Pen

The depletion of sulfadimidine and enrofloxacin (including its metabolite ciprofloxacin) is determined using the pigs in pen 1 of compartment 4 and the results are shown in Figure 1 for urine, manure and wipes of the treated pigs (*n* = 4; solid line) and untreated pigs (*n* = 4; dashed line) housed in the same pen. Pig A–D were placed in pen 1A (in black) and pig E–H in pen 1B (in grey). Pigs A and B, and pigs E and F, were treated. The time is the number of days after the start of the treatment (treatment was from day 0 to day 4). Concentrations in manure and urine are expressed in µg kg^−1^ and µg L^−1^, respectively. Wipe sample concentrations are expressed in nanograms per wipe. Note that the measured concentrations of pigs treated particularly with sulfadimidine were generally high. This was especially true of urine, where concentrations exceeded the calibration range (>10,000) with a factor between 2 and 10 for all treated animals, with the exception of one, on days 1, 4 and 7. As these concentrations were outside the calibration range, and dilution could not be carried out, the reported values are estimates based on extrapolation of the calibration curve (correlation coefficient (R) ≥ 0.990). For manure, extrapolated concentrations are indicated in the Supplementary Materials with a hashtag (#) (Figures S4–S6; compartment 4). For wipes, all residue levels are estimates based on a calibration curve with R ≥ 0.990.

The concentrations detected in manure and urine of the treated pigs are similar in order of magnitude for sulfadimidine (between 50,000 and 100,000 µg kg^−1^ or µg L^−1^ on day 4). On the other hand, a difference can be seen for enrofloxacin and ciprofloxacin; in manure, mainly enrofloxacin itself is excreted, while in urine, mainly the metabolite ciprofloxacin is excreted. Because of the differences in dosage, concentrations of sulfadimidine are higher than those of enrofloxacin and its metabolite. When reviewing all results of all matrices, it becomes clear that, in all cases, residues transfer from the treated animal to the untreated animal. For wipe samples, there is little difference between the residue levels detected on treated and untreated pigs. For the internal matrices, urine and manure the concentrations detected in the untreated animal are at least a factor 10 lower compared to the treated animals during treatment. However, after treatment, this difference in concentration quickly becomes smaller, and in all cases from 10 days after treatment, no distinction can be made between either the urine or the manure of treated and untreated pigs.

### 2.3. Transmission of Antibiotics to Internal Matrices of Untreated Pigs in Different Pens

Transmission of antibiotic residues to the internal matrices was determined through the analyses of manure (taken rectally). Figure 2 shows a descriptive summary of the sum of measured concentrations across sampling times of sulfadimidine, enrofloxacin and ciprofloxacin in manure, depicting transmission expressed as the relative residue level (%) in the internal matrix manure of untreated animals compared with the treated animals in pen 1. The error bar represents the average deviation between the duplicate pens (A and B) (see Section 4.9 for details on the calculations). The concentrations (µg kg^−1^) detected in manure per animal at each sampling time point are shown in Figures S4–S6 in the Supplementary Materials for sulfadimidine, enrofloxacin and ciprofloxacin. 

Figure 2 shows that ciprofloxacin was not detected above the LOQ in any of the untreated pigs. For enrofloxacin, no residues were detected above the LOQ in untreated pigs in compartments 1, 2 and 3. In compartment 4 (interconnected open pens), enrofloxacin was detected in pen 2, until day 10 (Figure S5). For sulfadimidine, no residues were found above the LOQ in the manure of untreated pigs in compartment 1 (separated closed pens). In compartment 2 (separated open pens), a single positive manure sample was found in one of the duplicates of pen 3 on day 7 (Figure S4). This result could not be explained. In compartment 3 (interconnected closed pens), residues were found in pen 2 on days 4, 7 and 10 and a single time in pen 3 on day 7. No sulfadimidine was detected above the LOQ in the manure of untreated pigs in pens 4 and 5 (furthest away from the treated pigs). In compartment 4 (interconnected open pens), sulfadimidine residues were detected in pen 2 on all days after the start of the treatment. Additionally, sulfadimidine residues were detected in pigs in pen 3 on days 7, 10 and 17. In some cases, sulfadimidine was also detected in the manure of animals kept in pens 4 (single pig on day 7) and 5 (single pig on day 7 and 10).

### 2.4. Transmission of Antibiotics to External Matrices of Untreated Pigs in Different Pens

In Figure 3, the sum of measured residue levels across sampling times (ng per wipe) of all sampling time points is visualized as a descriptive summary for sulfadimidine, enrofloxacin and ciprofloxacin determined in wipes of the pigs in the various compartments. The relative residue levels in wipes of untreated pigs compared to the levels in wipes of the treated pigs are displayed above each bar graph (%). The error bar represents the average deviation between the duplicate pens (A and B) (see Section 4.9 for details on the calculations). Additionally, a detailed overview of the results on each sampling day is provided in Figures S1–S3 in the Supplementary Materials for sulfadimidine, enrofloxacin and ciprofloxacin, respectively, mapping the transmission of residues over time. 

Figure 3 shows that, the farther away one moves from the pen with treated pigs, the smaller the percentage of transmission with each pen becomes. The spread of sulfadimidine and enrofloxacin appears more pronounced in compartment 2 (open partitions) compared to compartment 1 (closed partitions), although the difference should be interpreted with caution considering the larger variation between duplicate pens for compartment 2. Most transmission from treated to untreated pigs occurs in compartment 4 (interconnected pens with open partitions). The percentage of transmission for sulfadimidine is similar to the percentage of spread for enrofloxacin.

On day 17, wipe samples for two animals were accidentally omitted. This concerns one of the animals in pen 1 of compartment 2 and one of the animals in pen 2 of compartment 4. This is indicated with a “-” in Figures S1–S3. The results were omitted in the summation in order to create Figure 3. 

### 2.5. Transmission of Antibiotics to the Barn Environment

Figure 4 shows a descriptive summary of sulfadimidine, enrofloxacin and ciprofloxacin residues detected in the compartment, representing the barn environment, by means of hangers above the pens (Hs) and tripods (Ts) placed in the corners of the compartment, each with a round plateau to wipe. The Hs (hangers) and Ts (tripods) are shown schematically below the graphs to indicate the location. The results per time point are also included in Figures S1–S3 (Supplementary Materials).

The results of the environmental sampling in Figure 4 show that transmission of sulfadimidine and enrofloxacin residues occurs through the whole compartment; antibiotic residues spread not only to the tripods and hangers closest to the pen with treated pigs, but also to those farthest away, placed at the other side of the barn. When looking at the residue transmission patterns, compartments 1 (separated, closed pens) and 2 (separated, open pens) are comparable, while increased levels of residues to the plates above the pens are shown in compartment 3 (interconnected, closed pens), and a more gradual, spread-out residue pattern could be observed in compartment 4 (interconnected, open pens). Figures S1 and S2 show that residues are detected for sulfadimidine and enrofloxacin from day 1 after treatment, whereafter antibiotic residue levels reduce in the days after treatment. Nevertheless, antibiotic residues are still detected in the barn environment 2 weeks after the treatment. The level of enrofloxacin’s metabolite ciprofloxacin is at least 10 times lower and therefore faster to fall below the detection limit of the method (5 ng per wipe for ciprofloxacin). In compartments 1 and 2, transmission of ciprofloxacin residues (Figure S3) only occurred in the hangers above the pen with treated pigs and the tripods nearest to this pen. In compartments 3 and 4, transmission patterns are comparable to the residue transmission patterns of enrofloxacin and sulfadimidine.

## 3. Discussion

For pigs in the same pen, the transfer of antibiotic residues occurs from the treated pig to the untreated pig. For the external matrix wipes, the levels of antibiotic residues detected in untreated pigs are comparable to, or sometimes even higher than, the amount in treated pigs. This was also observed in wipes taken in a study with oxytetracycline in veal calves [13] and is probably caused by physical contact between the untreated pig and the treated pig and its contaminated manure and urine. Antibiotic residues were also detected in internal matrices (urine and manure) of the untreated pig, the latter being sampled rectally. The concentrations detected in the untreated animal are distinguishable (at least a factor 10 lower) compared to the treated animals during treatment, which was observed for other veterinary drugs in urine [15]. A possible explanation is that residues first need to be transferred from the treated to the untreated animal following contact. Higher excretion in the form of ciprofloxacin is seen in urine as opposed to enrofloxacin in manure, which is in line with depletion studies in pigs [16]. 

When investigating the transmission of antibiotic residues towards internal matrices of untreated pigs in other pens (Figure 2), concentrations in the untreated pigs were higher when contact with the treated pigs was possible (compartment 4 vs. compartments 1, 2 and 3). Under these experimental conditions, these findings suggest that restricting physical contact between treated and untreated pigs may be an important strategy for reducing antibiotic residues in internal matrices. With the observation of a small percentage of relative residue levels (of 0.04% for sulfadimidine) in compartment 3 (interconnected but closed pens) compared to no relative residue levels detected above LOQ for separated pens (compartments 1 and 2), separation seems less impactful in minimizing transmission of antibiotic residues than restricting physical contact. In previous research [7,11,12], where untreated pigs were housed in pens separated from the pen with treated pigs with space in between, external exposure (dust, air) was detected, but detection of internal exposure varied. Beyer et al. did not find any residues of ceftiofur (and metabolites) in plasma of the untreated pigs [11]. Janssen et al. did not find any residues of enrofloxacin or ciprofloxacin in plasma of untreated pigs, although low concentrations (nanogram range) were detected in urine [7]. Stahl et al. detected only traces of sulfadiazine in plasma (below LOQ), but high concentrations (mg L^−1^) in urine [12]. The pigs in the last study were, however, housed in a dry-cleaned barn where previously treated pigs were kept, which might cause more direct contact with contaminated surfaces. 

Differences between studies might be caused by the antibiotic used and by the treatment. Therefore, antibiotic type, the dosages of treatment and the LOQ of the analytical detection method are important to take into account. Based on the efficacy of the antibiotic used, the dosage for the sulfadimidine treatment in this study is 58 times higher compared to the enrofloxacin treatment. This results in higher concentrations of sulfadimidine residues in internal matrices and in the environment, which remain detectable for a longer duration. The detected concentrations for enrofloxacin and metabolite ciprofloxacin are lower, and might fall below the method’s limit of quantification (LOQ) more easily. The absence of ciprofloxacin residues in manure is most likely due to concentrations being too low to detect with the current analytical methods. It should also be noted that residues detected in rectal manure may also result from ingestion and gastrointestinal passage and therefore do not necessarily indicate systemic absorption.

When observing transmission of antibiotic residues to external matrices of untreated pigs in other pens (Figure 3) and the environment (Figure 4), it is apparent that there is a different degree of relative residue levels between compartment 4 (interconnected, open pens) and the other compartments due to physical contact between the pigs. This compartment also shows a more wide-spread residue transmission pattern to the environment (Figure 4). Transmission of antibiotic residues in general seems to occur in the same pattern whether using closed interconnected partitions (compartment 3) or separating the pens (compartments 1 and 2), which prevent direct contact between the pigs. Note that residue levels in wipes are estimated values (calculated using a calibration curve with R ≥ 0.990). The spatial distribution of residues suggests that airborne dust and/or aerosols may be important routes of external transmission from treated pigs to untreated pigs. 

The most probable modes of transmission of antibiotic residues through the air are (airborne) dust and aerosols, as these matrices are also carriers of bacteria originating from pig manure [11,17] and are known to harbour antibiotic residues [7,8,18], which can spread through the environment. 

A factor that might influence the transmission of antibiotic residues is the number of pigs in the compartments. Although the number of treated pigs is the same in each compartment, transmission to the environment seems higher in compartments 3 and 4, housing 24 pigs, compared to compartments 1 and 2, housing 16 pigs. The higher number of animals may result in more dust formation or air movement, in turn resulting in more airborne dust and a higher transmission. As pen configuration, compartment, and, to some extent, animal density varied concurrently, differences among compartments should therefore be interpreted with caution and considered exploratory rather than solely attributable to the specific partition types.

In this study, strict rules were in place for the animal caretakers in order not to influence the study results. Untreated pigs farthest away from the treated pigs were tended to and sampled first (from right to left), ensuring there was no contact after tending to the treated pigs. In a real barn situation, rules such as this would not be in place. Similarly, the barn where pigs are placed is probably not totally free of antibiotic residues, as they are known to be persistent in barn dust [8] and can cause detection of residues in untreated pigs through oral uptake [12]. Therefore, the results in this study presumably present the best-case scenario, and more transmission is likely to be seen in a barn in practice. 

In conclusion, after antibiotic treatment, transmission of residues occurs in the barn environment and the in untreated pigs housed in the same place. During treatment, fewer residues were observed in untreated pigs under the conditions of this study when physical contact with treated and untreated pigs was prevented by separating the pigs and by having space between the pens. This set-up also resulted in lower external residue levels, where the number of pigs housed could be a possible factor to take into account as well. The results of this research could be taken into account in a real barn situation, where the barn environment may act as a reservoir of antibiotic residues, which, based on previous studies, could contribute to conditions favouring the selection or maintenance of antimicrobial-resistant bacteria and resistance genes. 

## 4. Materials and Methods

### 4.1. Animal Study

An animal study was conducted following ethical approval by the Animal Test Committee (AVD40100202010546). The pigs had unlimited access to feed and drinking water. Within the experimental set-up, at least two pigs were placed together in one pen. The bedding consisted of sawdust and this was added regularly. In each pen, varying enrichment was provided for the pigs. Overall, the animals remained healthy throughout the study, and no animals had to be euthanized during the study. One animal had a minor umbilical hernia that did not result in complications and did not require treatment. On one occasion, an animal developed a leg lesion and was treated with antibiotics (ampicillin) and a non-steroidal anti-inflammatory drug (NSAID; meloxicam). These events were not considered to affect the study outcomes.

### 4.2. Experimental Design

For the study, 80 pigs (Sus scrofa domesticus, crossbreed finishing pigs; 40 males and 40 females), approximately 10 weeks of age and with a mean body weight of 25 kg, were obtained from an EU-based breeder. No information on previous antimicrobial treatment was available for the animals prior to the start of the study. Upon arrival, the pigs were given ear tags for identification and were distributed among the pens in four separate compartments (compartments 1–4), with an equal number of males and females in each pen. No randomization or blinding was applied. Each compartment contained different pen arrangements (Figure 5). The extent of physical contact between the pigs was varied by separating adjacent pens with open or closed partitions. Transmission of antibiotic residues was investigated by varying between open and closed partitions and by varying between interconnected pens and separate pens.

In each compartment, four pigs were housed in pen 1 and two pigs in the subsequent pens. In compartment 1, a total of twelve rectangular pens were set up in a row of six by two, where the two pens placed against each other on the short side served as duplicates. The duplicate pens were in all cases separated by a closed PVC partition. No physical contact could take place between the pigs in the duplicate pen due to the height of the partition (approximately 1.2 m). The pens in compartment 1 were set up separated, with a space in between, and closed partitions were used, which consisted of the same PVC plates as were used as partition between the duplicate pens. Compartment 2 had the same layout as compartment 1, but the partitions of the pens consisted of fencing, leaving three sides of the pens open. In compartment 3, the pens were arranged interconnected to each other, with closed PVC plates covering all sides of the pen, similar to the pens in compartment 1. Compartment 4 consisted of interconnected pens with open fencing as a partition, similar to the pens in compartment 2. 

The number of pigs was determined by the experimental design and the required number of experimental units. Each experimental unit required at least two treated and two untreated pigs housed within the same pen. In addition, at least two pens containing untreated pigs were included in each compartment, with the most distant pen at roughly the same distance as the treated animals from the last interconnected pen. This resulted in five interconnected pens per compartment. To further assess transmission patterns, a duplicate set of pens at the top and bottom of the compartments was included. Based on this design, a total of 80 pigs were required for the study.

In all compartments, tripods (T, ±70 cm high) and hangers (H, placed ±200 cm high) with round plateaus (9 cm diameter) were set up for taking environmental wipe samples (dark dots in Figure 5). Tripods were placed in the four corners of the compartment (indicated with T), and hangers (indicated with H) were placed in the middle, above each pen duplicate.

### 4.3. Treatment

Pigs were treated (one male and one female per pen) with sulfadimidine and enrofloxacin. These two antibiotics are not often used in the treatment of pigs, reducing the chance of contaminating the pigs and the barn environment at the start of the study. 

Treatments were administered orally to assume a worst-case scenario, as oral administration is expected to result in a greater degree of spreading compared to injection. To prevent variation in administered dose, antibiotics were administered to the animal directly and not via the drinking water. 

The first week (day −7 to day −1) was used to acclimatize the pigs; no treatment was carried out. In the second week, two of the four pigs in pen 1 (in all compartments) were treated with 1.7 mg kg^−1^ body weight Enrox 100 mg mL^−1^ (REG NL 10409, KRKA, d.d., Novo mesto, Slovenia) and 100 mg kg^−1^ body weight Sulfadimidine-Na (REG NL 1311, Dopharma Research B.V., Raamsdonksveer, The Netherlands) for 5 consecutive days (day 0–4). The Enrox formulation for chickens and turkeys was used because the formulation for pigs (Enrotron 5 mg mL^−1^, REG NL 110979, aniMedica GmbH, Senden-Bösensell, Germany) was not available. The dosage administered (1.7 mg kg^−1^ body weight) corresponded to the dosage specified for pigs in REG NL 110979. The number of days represents the number of days after the start of treatment. The entire animal study lasted 4 weeks (day −7 to day 20), taking into account the long withdrawal period for sulfadimidine (15 days).

### 4.4. Sampling

A great effort was made to eliminate possible contamination that would influence the results of the study. Therefore, after the compartments and pens were set up, but before the pigs arrived, a total of 84 wipe samples were taken from the PVC plates, fencing, feeding system and pipes above the pens. Wipes were sampled by taking a moist ethanol wipe (Cemex B.V., Bleiswijk, The Netherlands), wiping it over a large surface and placing it into a 50 mL PP tube (Greiner Bio-One, Alphen aan den Rijn, The Netherlands). All wipes were analyzed for sulfadimidine, enrofloxacin and ciprofloxacin residues before the animals were placed in the compartments.

The compartments were separated spaces in the same building, with their own entrance and exit. To avoid possible cross-contamination during the handling of the animals by the caretakers, a one-way walking route and strict clothing changing instructions were in place. Sampling started at the right end of the compartment, farthest away from the treated animals. For each of the compartments, new disposable overalls and hair nets were taken. For each pen, there was a different set of boots. For each separate sampling, new gloves were used. Sampling ended at the leftmost pen, which housed both treated and untreated animals, where the treated animals were sampled last. The caretakers left the compartment on the left side and disposed of their overalls. This procedure was repeated for all four compartments at each sampling time point. 

There were a total of seven sampling time points: day −4, day 1, day 4, day 7, day 10, day 14 and day 17. At each time point, a wipe sample of the pigs, wipe samples of the environment (tripods and hangers) and a manure sample were taken. Manure was taken rectally in order to determine the degree of internal residue presence. Urine samples were collected from pigs in pen 1 of compartment 4 on days 1, 4, 7, 10, 14, and 17 to compare depletion patterns with those observed in manure and wipe samples. Urine was collected by manually positioning a collection container beneath the pig during spontaneous urination, with a target volume of at least 100–200 mL per sample. Wipes of the pigs were sampled by taking a moist ethanol wipe and wiping it over the middle of the back of the pig. This way, it was easier to always sample at the same location at each time point. Environmental wipes were sampled by wiping the entire surface of the plateaus of the tripods and hangers. The wipe was then immediately placed in a 50 mL PP tube. All samples were stored at −18 °C until analysis.

### 4.5. Reference Standards

Enrofloxacin, ciprofloxacin and sulfadimidine were ordered from Sigma Aldrich (St. Louis, MO, USA). In addition, the internal standards sulfadimidine-^13^C_6_, enrofloxacin-d_5_ and ciprofloxacin-d_8_ hydrochloride sesquihydrate were ordered from Witega (Berlin, Germany) to correct for variations in sample preparation and LC-MS/MS analysis. Stock solutions of the reference standards and internal standards were prepared at 1000 mg L^−1^ in methanol. A mixed solution of reference standards was prepared at 10 mg L^−1^, 2.5 mg L^−1^ and 0.1 mg L^−1^ in methanol. A mixed solution of internal standards was prepared at 5 and 10 mg L^−1^ in methanol.

### 4.6. Solvents and Reagents

Methanol ULC/MS grade and acetonitrile ULC/MS grade were purchased from Actu-All Chemicals (Oss, The Netherlands). Formic acid (FA), citric acid monohydrate, sodium hydroxide (NaOH), and disodium hydrogen phosphate dihydrate were purchased at VWR International (Darmstadt, Germany). Ammonium hydroxide (25%) was purchased at Merck Millipore (Darmstadt, Germany). Trifluoroacetic acid (TFA) was purchased at Sigma-Aldrich. Milli-Q water (water) was prepared using a Milli-Q system with a resistivity of at least 18.2 mΩ.cm (Merck Millipore). 

McIlvain-ethylenediaminetetraacetic acid (EDTA) buffer was prepared by dissolving 74.4 g EDTA disodium (Titriplex^®^ III, VWR International, Darmstadt, Germany) into 500 mL of 0.1 M citric acid (citric acid monohydrate, VWR International) and 280 mL of 0.2 M phosphate buffer (disodium hydrogen phosphate dihydrate, VWR International). Then, 1 L of MilliQ water was added, whereafter the pH was adjusted to 4.0 (if necessary) by adding 0.1 M citric acid or 0.2 M phosphate buffer. Finally, the total volume was adjusted to 2 L with MilliQ water.

### 4.7. Sample Preparation

Manure samples were extracted according to [19]; 2 g of manure was extracted using 4 mL of 0.125% TFA in acetonitrile and subsequently 4 mL of McIlvain EDTA buffer. After adding 2 mL of lead acetate solution (200 g L^−1^), samples were centrifuged. The supernatant was decanted into a glass tube and the acetonitrile was evaporated under a nitrogen stream at 40 °C. After evaporation of the acetonitrile, 13 mL of 0.2 M EDTA solution was added to the remaining extract. Wipe samples were extracted according to [20]: wipes were processed entirely. After opening the tubes to let the wipes dry, they were extracted using 15 mL of McIlvain EDTA buffer and centrifuged at 3500× *g* during 10 min. Urine samples were prepared by extracting 1 mL of urine with 2 mL of McIlvain EDTA buffer. After addition of the buffer, the pH was checked and, if necessary, adjusted to 4.0 with 37% hydrochloric acid. For all matrices, solid phase extraction clean-up was carried out by first conditioning a reversed phase 6 mL, 200 mg SPE cartridge (Strata-X, Phenomenex, Torrance, CA, USA) with 5 mL methanol and 5 mL McIlvain-EDTA buffer subsequently. Then, the entire extract was brought onto the cartridge. After rinsing with 5 mL of water, extracts were eluted with 5 mL of methanol. This final extract was brought into a vial and injected into the LC-MS/MS system.

### 4.8. LC-MS/MS Analysis

Analysis was carried out using the UHPLC-MS/MS settings according to Jansen et al. [20]. The settings for compound-specific parameters Scheduled Reaction Monitoring (SRM) transitions, declustering potential (DP), collision energy (CE) and Collision Cell Exit Potential (CXP) are shown in Table 1. The entrance potential (EP) was in all cases set to 10.

### 4.9. Calculations

For the preparation of the descriptive summaries in Figure 2 and Figure 3, transmission was expressed as the relative residue level (%) in untreated animals compared with treated animals for the internal (manure) and external (animal wipes) matrices. The measured concentrations across six sampling time points (days 1, 4, 7, 10, 14 and 17) were summed for the two animals within each replicate pen (pen a and b summed separately). For pen 1, treated and untreated animals were summed separately. For each group, the mean summed concentration was then calculated from the two replicate pens (a and b). For pen 1, the mean was calculated separately for treated and untreated animals. The error bars represent the average deviation between the summed concentrations of pens a and b. Relative residue level (%) was calculated by dividing the mean summed concentration in untreated animals by the mean summed concentration in treated animals and multiplying by 100.

For environmental wipes (Figure 4), the residue levels measured for the tripods in the corners of the compartment were summed separately, after which, the mean residue level for the left and right was calculated and is presented as a mean in Figure 4. The error bars represent the average deviation between the summed concentrations from the top and bottom tripods. Note that the summary of the observed residue levels is intended as a descriptive measure and is not interpreted as an estimate of total exposure. Similarly, transmission is used as an operational metric and does not represent a mass-balance estimate.

### 4.10. Method Validation and Quality Control

The following validation criteria were determined for enrofloxacin, ciprofloxacin and sulfadimidine: selectivity, the limit of quantification (LOQ) and confirmation of identity. The selectivity was determined by evaluating whether blank samples, without addition of reference standards, contained interfering signals at the retention time of the analyte. Confirmation of identity was according to (EU) 2021/808, whereby the average retention time and relative ion ratio of the compounds present in the matrix fortified calibration line were determined. The maximum relative deviation of the ion ratio of the compounds in the samples should be within ± 40% and the maximum retention time deviation should not be larger than 0.1 min. The limit of quantification (LOQ) was determined at the concentration where the signal-to-noise ratio of the least abundant product ion was at least 6. Signals detected in samples below the LOQ therefore automatically did not meet the confirmation criteria. In general, samples containing signals that did not meet the confirmation criteria were excluded from further consideration and assigned a value of zero in the dataset for subsequent calculations.

To provide an estimation of the concentration in the samples that was as accurate as possible, matrix fortified calibration lines were prepared by fortifying blank matrix with compounds. Concentrations ranged from 0 to 100 ng per wipe, from 0 to 10,000 µg kg^−1^ and from 0 to 10,000 µg L^−1^ for wipes, manure and urine, respectively. As blank material for wipes, clean wipes were taken from the package. For manure and urine, in-house pig samples that were previously tested blank for the target analytes were used. Furthermore, the calibration line and all samples were fortified with stable isotope-labelled compounds (sulfadimidine-^13^C_6_, enrofloxacin-d_5_ and ciprofloxacin-d_8_) to ensure correction of losses in recovery and matrix effects. The calibration curves had a correlation coefficient (R) criterion of R ≥ 0.990. In case concentrations were found in the samples above the highest calibration point, results were extrapolated. All results reported comply with the confirmation criteria as stated in (EU) 2021/808 [14].

Quantitative parameters were previously determined in the in-house method validation for urine and manure. However, it should be noted that the urine method was only validated using bovine urine. Six samples were spiked at 50, 100, and 150 µg L^−1^ with enrofloxacin, ciprofloxacin, and sulfadimidine. Acceptance criteria for trueness were 80–120%, and for reproducibility (CV), 25% at 50 and 100 µg L^−1^ and 22% at 150 µg L^−1^. The previously published method for manure [19] was additionally validated using pig manure. Six blank pig manure samples were spiked at 20 µg kg^−1^ for enrofloxacin and ciprofloxacin and at 5 µg kg^−1^ for sulfadimidine. Acceptance criteria for trueness were 80–120% for enrofloxacin and ciprofloxacin and 70–120% for sulfadimidine, with reproducibility (CV) criteria of 25% and 30%, respectively. No quantitative parameters were determined for wipe samples; therefore, concentrations are reported as estimates in ng per wipe.

## Figures and Tables

**Figure 1:**
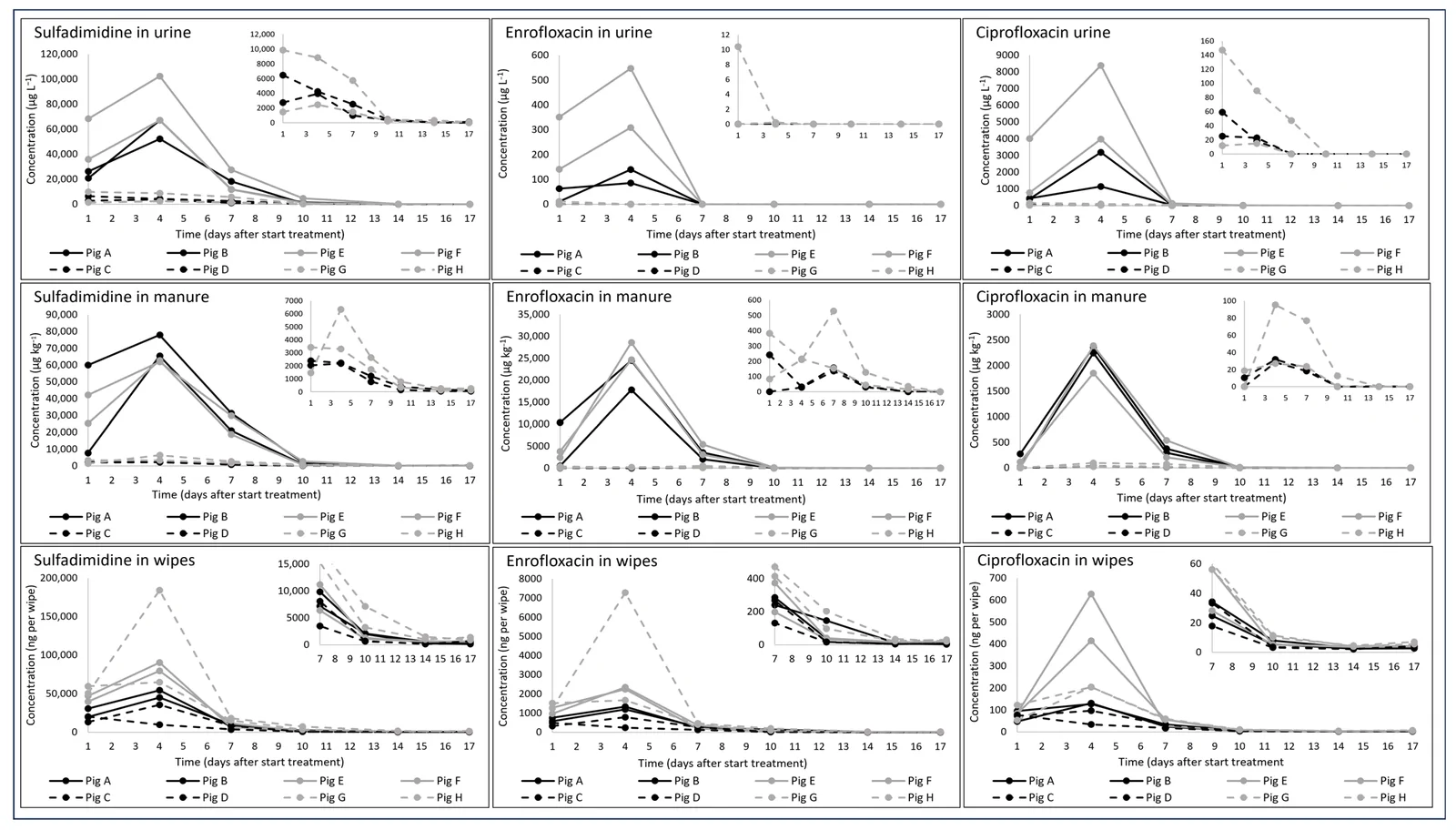
The concentrations of sulfadimidine, enrofloxacin and ciprofloxacin over time in manure (µg kg^−1^), urine (µg L^−1^) and wipes (ng per wipe). Pigs A–D were placed in pen 1A (black) and pigs E–H were in pen 1B (grey), of which 2 pigs per pen were treated (solid line) and 2 pigs were untreated (dashed line). The time in days is the number of days after the start of treatment. The scale on the *y*-axis varies per graph. The insert in the right corner shows a magnified view of the lower concentrations. For concentrations in manure and urine >10,000, concentrations are estimated using a calibration curve. For wipes, all residue levels were estimated using a calibration curve (R ≥ 0.990).

**Figure 2:**
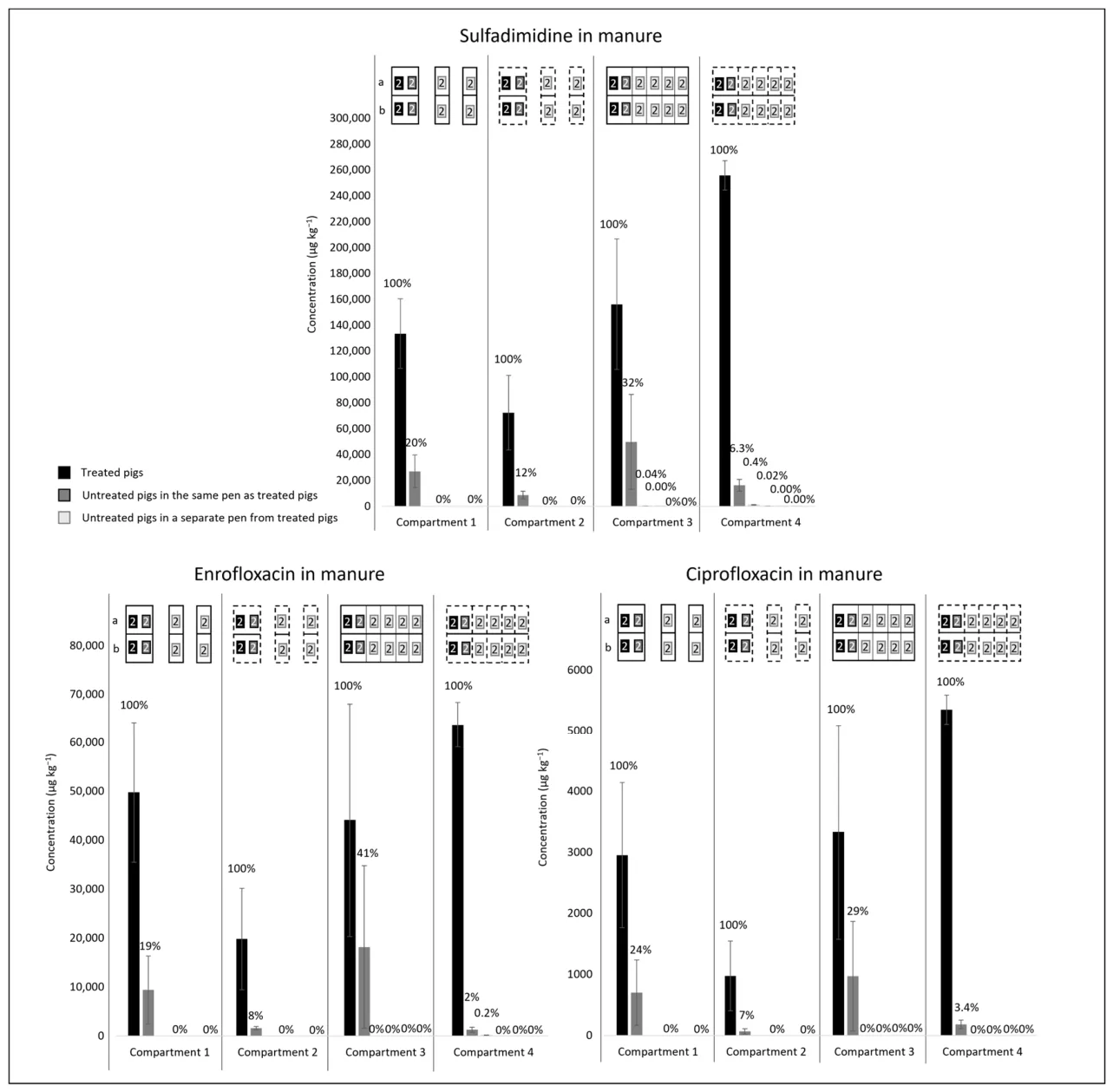
Sum of measured concentrations across sampling times of sulfadimidine, enrofloxacin, and the metabolite ciprofloxacin in pig manure in the various compartments. The layout of the compartments is shown schematically above, where pen 1 contains 2 treated (black) and 2 untreated pigs (dark grey) and the other pens all contain untreated pigs (light grey). The solid lines represent closed partitions and the dashed lines represent the fences with open partitions. The error bar represents the average deviation between the duplicate pens (a and b). Above the bar graph, the percentage of transmission is shown relative to the treated pigs in pen 1 (*n* = 2, black). Concentrations >10,000 are extrapolated estimates based on a calibration curve with R ≥ 0.990.

**Figure 3:**
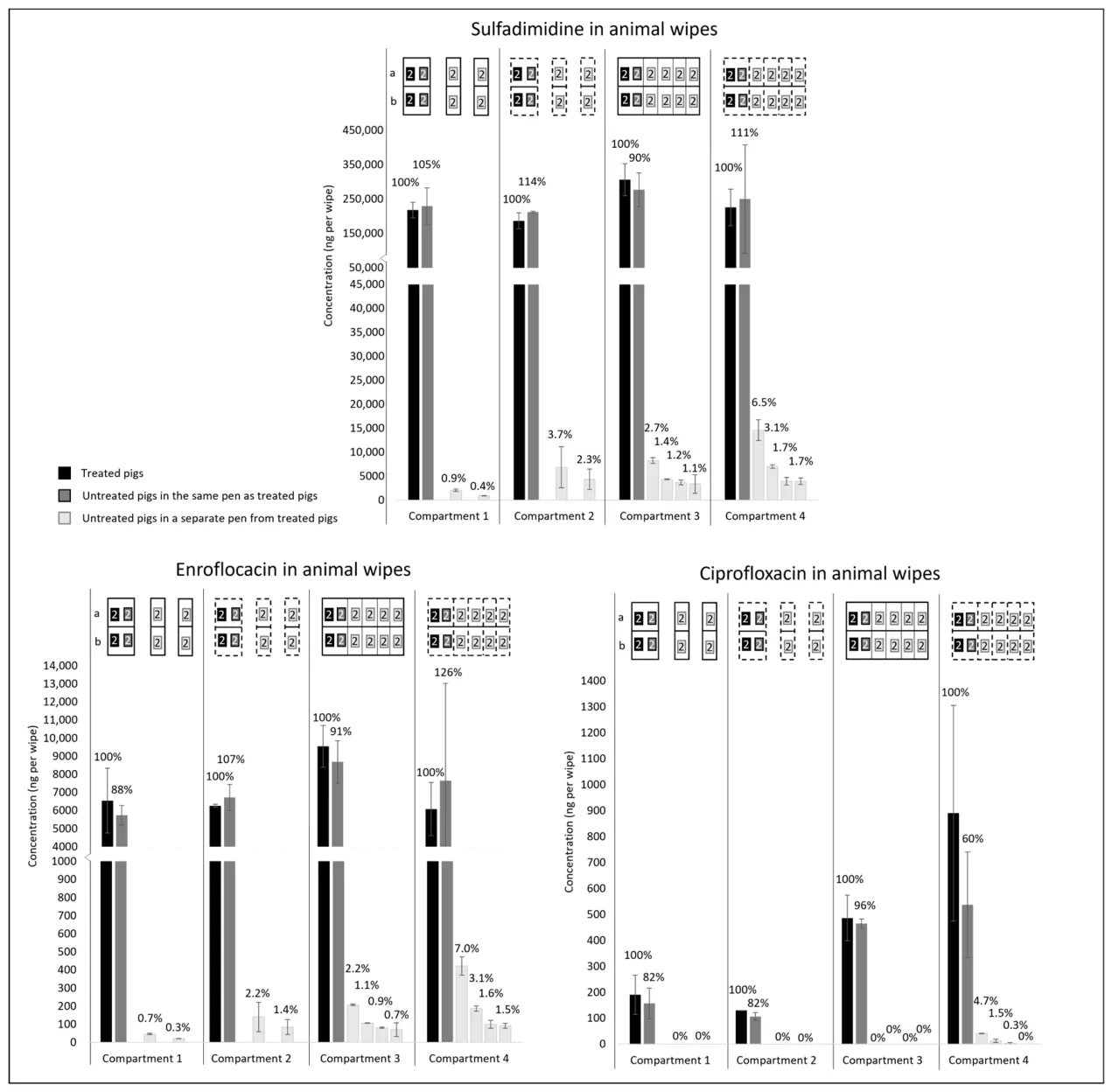
Sum of measured residue levels across sampling times of sulfadimidine, enrofloxacin, and the metabolite ciprofloxacin in animal wipes of the pigs in the various compartments. The layout of the compartments is shown schematically above, where pen 1 contains 2 treated (black) and 2 untreated pigs (dark grey) and the other pens all contain untreated pigs (light grey). The solid lines represent closed partitions and the dashed lines represent the fences with open partitions. The error bar represents the average deviation between the duplicate pens (a and b). Above the bar graph, the percentage of transmission is shown relative to the treated pigs in pen 1 (*n* = 2, black). Residue levels were estimated using a calibration curve (R ≥ 0.990).

**Figure 4:**
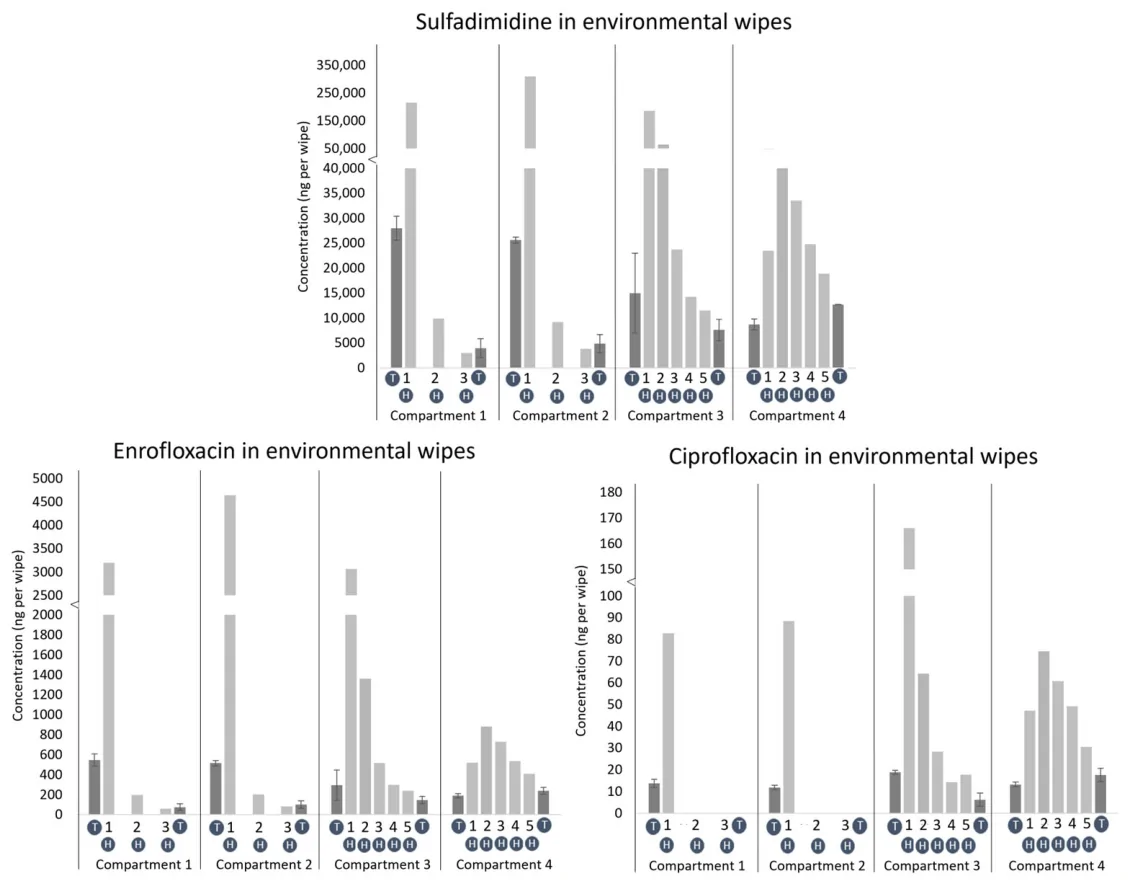
The sum of measured residue levels across sampling times of sulfadimidine, enrofloxacin, and ciprofloxacin in wipes of tripods (Ts) placed in the four corners of the compartments and in one hanger (H) that was placed in the middle above each pen. The symbols are shown schematically below the graphs. The error bars in the images represent the variation between the tripods (top and bottom corners of the compartment) positioned to the left and right of the barn. There are no duplicates for the hangers. H1 corresponds to the hanger above the pen with treated pigs. Residue levels were estimated using a calibration curve (R ≥ 0.990).

**Figure 5:**
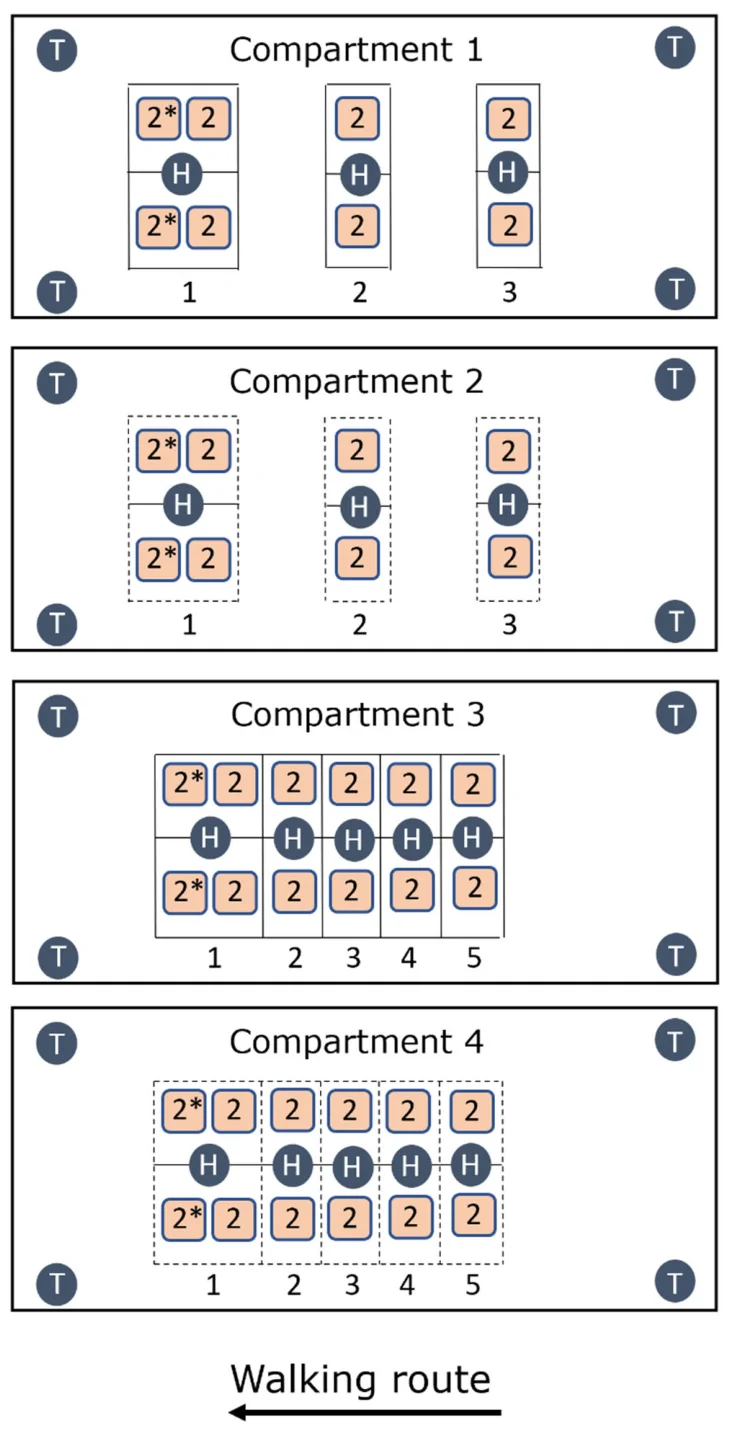
Schematic representation of the different compartments (1–4), where the solid lines represent closed partitions and the dashed lines represent the fences with open partitions. The orange boxes with numbers indicate the number of pigs in the pen, where numbers with an asterisk (*) indicate the treated animals. The dark dots in the corners are tripods (T1–4) with a plastic plateau on top. The dark dots in the middle of the pens are hangers with similar plastic plateaus (H1–5).

**Table 1:** The compound-specific settings in the LC-MS/MS method.

Compound ID	Q1 Mass (Da)	Q3 Mass (Da)	Declustering Potential (Volts)	Collision Energy (Volts)	Collision Cell Exit Potential (Volts)
Ciprofloxacin-d_8_	340.1	235.0	50	51	18
Ciprofloxacin	332.0	231.0	66	49	20
		288.0	66	25	22
Enrofloxacin-d_5_	365.1	321.0	56	27	22
Enrofloxacin	360.1	286.0	61	47	22
		316.0	61	27	24
Sulfadimidine	279.0	124.0	66	31	8
		186.0	66	23	16
Sulfadimidine-^13^C_6_	285.0	185.9	66	23	16

## Data Availability

The original contributions presented in this study are included in the article/Supplementary Material. Further inquiries can be directed to the corresponding author.

## Supplementary Materials

The following supporting information can be downloaded at: https://www.mdpi.com/article/10.3390/antibiotics15100977/s1, Figure S1: Sulfadimidine in ng per wipe in surface wipes of hangers (Hs) and tripods (Ts) and pig wipes per time point per compartment; Figure S2: Enrofloxacin in ng per wipe in surface wipes of hangers (Hs) and tripods (Ts) and pig wipes per time point per compartment; Figure S3: Ciprofloxacin (metabolite of enrofloxacin) in ng per wipe in surface wipes of hangers (Hs) and tripods (Ts) and pig wipes per time point per compartment; Figure S4: Sulfadimidine in µg kg^−1^ in pig manure per time point per compartment; Figure S5: Enrofloxacin in µg kg^−1^ in pig manure per time point per compartment; Figure S6: Ciprofloxacin (metabolite of enrofloxacin) in µg kg^−1^ in pig manure per time point per compartment.
